# Increasing Uptake of Long-Acting Reversible Contraceptives in Cambodia Through a Voucher Program: Evidence From a Difference-in-Differences Analysis

**DOI:** 10.9745/GHSP-D-16-00083

**Published:** 2016-08-11

**Authors:** Ashish Bajracharya, Lo Veasnakiry, Tung Rathavy, Ben Bellows

**Affiliations:** a Population Council, Phnom Penh, Cambodia; b Ministry of Health, Department of Public Health and Information, Phnom Penh, Cambodia; c Ministry of Health, National Maternal and Child Health Center, Phnom Penh, Cambodia; d Population Council, Lusaka, Zambia

## Abstract

**Objective:**

This article evaluates the use of modern contraceptives among poor women exposed to a family planning voucher program in Cambodia, with a particular focus on the uptake of long-acting reversible contraceptives (LARCs).

**Methods:**

We used a quasi-experimental study design and data from before-and-after intervention cross-sectional household surveys (conducted in 2011 and 2013) in 9 voucher program districts in Kampong Thom, Kampot, and Prey Veng provinces, as well as 9 comparison districts in neighboring provinces, to evaluate changes in use of modern contraceptives and particularly LARCs in the 12 months preceding each survey. Survey participants in the analytical sample were currently married, non-pregnant women ages 18 to 45 years (N = 1,936 at baseline; N = 1,986 at endline). Difference-in-differences (DID) analyses were used to examine the impact of the family planning voucher.

**Results:**

Modern contraceptive use increased in both intervention and control areas between baseline and endline: in intervention areas, from 22.4% to 31.6%, and in control areas, from 25.2% to 31.0%. LARC use also increased significantly between baseline and endline in both intervention (from 1.4% to 6.7%) and control (from 1.9% to 3.5%) areas, but the increase in LARC use was 3.7 percentage points greater in the intervention area than in the control area (*P* = .002), suggesting a positive and significant association of the voucher program with LARC use. The greatest increases occurred among the poorest and least educated women.

**Conclusion:**

A family planning voucher program can increase access to and use of more effective long-acting methods among the poor by reducing financial and information barriers.

## Abstract (precis)

By reducing financial and information barriers, a family planning voucher program in Cambodia significantly increased contraceptive choice and uptake of more effective long-acting reversible contraceptives among poor women and women with the least education. Without vouchers, many of these women would not have used contraception or would not have chosen their preferred method.

## INTRODUCTION

From 2000 to 2010, contraceptive prevalence among married women of reproductive age in Cambodia increased dramatically and rapidly—from 24% in 2000 to 40% in 2005 and 51% in 2010.^1^ By 2010, knowledge of contraceptives methods among women was nearly universal.

Despite these gains, the use of modern methods, in particular long-acting reversible contraceptives (LARCs) and permanent methods, remained quite low. In 2010, 35% of married women were using modern methods, but only about 17% of these women were using LARCs or permanent methods.^1^ By comparison, 15% of modern method users were using the oral pill and 10% were using injectables. At the same time, more than half of married women said that they did not want more children or they wanted to space births by 2 years or more, and 17% of women in 2010 expressed an unmet need for contraception.^1^

Access to a full range of modern contraceptives contributes importantly to reductions in maternal mortality and morbidity, a key development goal in low- and middle-income countries.^2^ In most developing countries, use of short-acting modern contraceptives has greatly increased in response to family planning program initiatives. Significant inequities and disparities remain, however, in women’s access to highly effective LARCs.^3^^–^^6^

LARCs remain effective for years, enabling women to delay, space, or limit births as they choose,^2^ without the need for resupply that can be disrupted by failures in the supply chain. Although initial costs of LARCs are higher, the average cost over the period of use is often lower than that of less effective short-acting methods.^7^ However, these long-acting methods are often out of reach of the most vulnerable and marginalized women due to cost and gatekeeping by providers.^8^

As in many other low- and middle-income countries, in Cambodia barriers to LARCs exist at the patient, facility, health systems, and policy levels.^9^ While system-level barriers can present significant challenges to LARC uptake, high costs to the user, providers’ attitudes, and misinformation present the most significant obstacles to increasing access to and use of LARCs.^2^^,^^8^^–^^11^

High costs, providers’ attitudes, and misinformation present the most significant obstacles to increasing use of LARCs.

Subsidizing services and providing information for potential users who might otherwise be unable to use the service are essential to address these challenges. Studies of demand-side strategies such as vouchers, particularly vouchers for maternal, sexual, and reproductive health care, have found some increases in service utilization, particularly among low-income and marginalized groups.^12^^–^^15^ In Cambodia, the Reproductive Health (RH) Voucher program seeks to increase poor women’s access to maternal and reproductive health services and to increase uptake and expand choice of family planning methods. Although maternal health care voucher programs in Cambodia have been studied,^16^^,^^17^ no studies have reported on the effects of the family planning voucher under the RH Voucher program in Cambodia on uptake of modern contraceptives. In this article, we present analysis from an evaluation of the family planning voucher component of the RH Voucher program.

## PROGRAM DESCRIPTION

### The Cambodian Reproductive Health Voucher Program

The Cambodian Ministry of Health, with technical support from development partners, launched the RH Voucher program in 2010. The Voucher Management Agency (VMA), a technical group comprising EPOS Health Consultants, Oxford Policy Management, PriceWaterhouseCoopers, and Action for Health (AFH), managed and implemented the program. Funding came from the German Development Bank (KfW). The Ministry of Health and VMA designed the program and selected operational districts for participation after a number of formative studies, including rapid situational analyses, stakeholder consultations, and needs assessments conducted by VMA with assistance from international experts on voucher programs. The RH Voucher program complements the flagship Health Equity Fund (HEF) program, a strategy to improve access to health care for the poor.^18^ The RH voucher scheme offers vouchers for maternal health care and family planning in eligible, accredited public facilities and for safe abortion services in participating private facilities.

The RH Voucher pilot project took place in 110 health centers and their catchment areas in 9 operational districts in 3 Cambodian provinces: Kampong Thom, Kampot, and Prey Veng. Health centers were selected based on a concentration of poor residents in surrounding areas, and the health centers were accredited to implement the voucher program based on satisfactory scores on relevant components of a national quality assessment tool. Women were eligible for the voucher program if they held IDPoor cards (a poverty grading tool that pre-identifies beneficiaries for the national HEF program). If they had not been assessed under the IDPoor program, the RH Voucher program could conduct post-identification through a comparable grading tool.

Vouchers were promoted and distributed through community-based awareness-raising sessions, marketing campaigns, and face-to-face counseling by voucher promoters who informed eligible beneficiaries of the benefits of the services that the vouchers covered. Bellows et al. (2011) provide a full description of the RH Voucher program.^19^

### The Family Planning Voucher Component

Family planning vouchers, one component of the RH Voucher program, provided free access to any modern contraceptive, including short-acting methods, LARCs, and permanent methods. As part of the means-tested voucher program, community-based distributors identified eligible women of reproductive age holding IDPoor cards and provided them with the vouchers. Interested eligible women received comprehensive family planning counseling at a facility, typically a primary health center, and were given information on a comprehensive set of contraceptive methods. Clients who chose short-acting modern methods received the service at the primary health center. The health centers referred clients who selected LARCs or permanent methods to higher-level facilities where these methods were available. The voucher entitled beneficiaries to receive the method of their choice, including referral, at no cost. Beneficiaries also received a transportation subsidy of 500 riels (approximately US$0.13) per kilometer, including for referral if necessary.

## METHODS

The Population Council conducted an evaluation of the family planning voucher component of the RH Voucher program, with funding from the Bill and Melinda Gates Foundation, as part of a broader evaluation of voucher and accreditation programs in 5 countries in Africa and Asia. Our analysis focuses on the average effect of exposure to the voucher program (by living in voucher catchment areas) on net change in the use of modern contraceptives, with a focus on the uptake of LARCs. The family planning voucher was not specifically focused on increasing LARC uptake, but we hypothesized that it might improve the uptake of LARCs by reducing cost barriers by offering a free service with transportation and referral subsidies and by removing information barriers through comprehensive counseling.

### Study Design and Data

Our findings come from a quasi-experimental pre- and post-intervention study of the Cambodian RH Voucher Program conducted between 2011 and 2013 that used a mixed methodology that included household surveys, health facility assessments, interviews of clients and providers, and observations of client-provider interactions. For details of the research design, including sampling selection, sample size calculations, and matching of intervention and control sites, please see Bellows et al. (2011).^19^

The data used in this analysis come from cross-sectional baseline (2011) and endline (2013) household surveys conducted in 9 pilot voucher program operational districts in Kampong Thom, Kampot, and Prey Veng provinces, as well as in 9 comparison operational districts in neighboring provinces. Both surveys interviewed a total of 2,200 women and 800 men from households within a 5-km radius of contracted facilities and, similarly, in a 5-km radius of comparison facilities. Sample sizes were based on minimum detectable effect calculations that are detailed in the study protocol.^19^ We selected the 9 comparison operational districts by using propensity score matching of a number of facility-level characteristics including facility ownership, size, level of obstetric care, and characteristics of the population in the facilities’ catchment areas.^19^ The baseline survey was conducted in early 2011, before the vouchers were introduced to intervention areas. The endline survey was completed in mid-2013, after an 18-month intervention period between surveys. The RH Voucher program continues to function in the intervention areas after the collection of the endline data. Voucher programs did not operate in comparison areas at any time during the implementation of the evaluation.

The Population Council’s Institutional Review Board and the Cambodian National Ethics Committee for Health Research granted ethical approval for this study. All participants gave their informed consent before participating.

Participants in our analytical sample were currently married, non-pregnant women ages 18 to 45 years (N = 1,936 at baseline and N = 1,986 at endline) who had answered questions in the baseline and endline surveys on contraceptive use and for whom data were available on key indicators used in the analysis. The Center for Advanced Studies (CAS) Cambodia, in collaboration with the Population Council, collected the data.

### Key Measures

The primary outcome variable was use of modern contraceptives among currently married women of reproductive age in the 12 months preceding each survey. We coded contraceptive use as a categorical variable for a comprehensive set of contraceptives. The 5 contraceptive use outcomes in this study are: (1) non-use of contraception, (2) use of traditional methods, (3) use of short-acting modern methods, (4) use of LARCs, and (5) use of permanent methods. A woman was recorded as using a short-acting modern contraceptive if she reported using condoms, oral pills, or injectables as her primary method. We coded women as using a LARC if they reported any use of an intrauterine device (IUD) or a hormonal implant in the 12 months before the survey. Women were recorded as using a permanent method if they had ever had a female sterilization procedure performed or if their spouse or partner had had a vasectomy. We coded any use of withdrawal or safe days as use of a traditional method.

The core analysis in this study involves measuring the association of exposure to the family planning voucher with uptake of various types of contraceptive methods—LARCs, short-acting modern methods, permanent methods, and traditional methods. We considered respondents to be exposed to vouchers if they lived within 5 km of a facility accredited by the voucher program.

The study presents results of an intent-to-treat analysis of the effect of the family planning voucher on uptake of contraceptives, thus comparing differences in uptake between respondents living in areas where the voucher program operated and respondents living where the voucher program did not operate (as contrasted with a comparison between individuals who used a voucher and those who did not). Thus, women need not have reported use of the voucher to be considered an intervention area participant. Participants from comparison sites were considered to have had no exposure to the voucher program.

The surveys collected data on a range of sociodemographic indicators, including women’s age, parity, educational attainment, occupation, religion, and socioeconomic status. We coded parity as no children; 1 child; 2 children; or 3 or more children. We categorized educational attainment as no schooling; completed primary school; completed secondary school (up to grade 9); or high school (grades 10–12) and higher level of education. We coded religion as Buddhism or other. We estimated socioeconomic status using household asset-based wealth quintiles constructed using principal components methodology devised by Filmer and Pritchett,^20^ which is also used in Demographic and Health Surveys to measure socioeconomic status.

### Data Analysis: Empirical Strategy

We present 2 sets of analyses in this paper. First, we present descriptive analysis of the sociodemographic characteristics of women as well as contraceptive use and method mix in both intervention and comparison samples at baseline. Second, we test statistical associations between voucher exposure and net change in contraceptive use, using the difference-in-differences (DID) technique^21^^,^^22^ to determine whether changes in LARC uptake and in uptake of other contraceptives are associated with women’s exposure to the family planning voucher. The estimation can be represented by a simple equation:

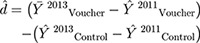


The estimate *d̂* measures the net change attributable to the intervention by ascertaining the difference between changes in the use of modern contraceptives (represented by *Y̅*, indicating the proportion of use), and specifically LARCs, for women in the voucher intervention and control areas before the intervention and 18 months later. To estimate a net effect, any observed change in the control areas in the use of modern contraceptives generally or in the use of LARCs specifically cannot be attributed to the voucher intervention and thus must be subtracted from the change observed in intervention areas. A key assumption of DID estimation is that preexisting outcome trends between intervention and comparison groups were similar.

We used *t* tests to gauge the significance for the DID estimates as well as for other appropriate 2-sample test analyses. All data analyses were conducted using STATA 13.

## RESULTS

### Background Characteristics of Women

In Table 1, we present descriptive statistics from the full sample in the baseline as well as from the intervention and control samples. There are no significant variations between the intervention and control samples in the distribution of most sociodemographic characteristics. The only statistically significant difference is in socioeconomic status. The control group was slightly wealthier than the intervention group. It is likely that this difference is a result of a higher proportion of poor women living near contracted voucher facilities in the intervention areas.

**TABLE 1.** Percentage Distribution of Women by Sociodemographic Characteristics in Baseline Survey, Cambodia, 2011

	Full Sample (N = 1,936)	Voucher Areas (N = 961)	Non-Voucher Areas (N = 975)	*P* Value
Age, years, mean	29.2	29.4	29.1	
15–19	2.4	2.5	2.3	.84
20–24	22.1	19.9	24.3	.02
25–29	31.8	32.0	31.5	.79
30–34	23.4	24.6	22.3	.23
35–39	11.8	13.8	9.9	.007
40–45	8.5	7.2	9.7	.04
Education level				
No school	17.4	18.0	16.7	.46
Primary school	58.8	59.2	58.5	.74
Secondary school (up to grade 9)	20.3	18.8	21.6	.13
High school (grades 10–12) or higher	3.5	4.0	3.2	.36
Occupational status				
Unemployed	12.5	13.2	11.8	.35
Agriculture	61.3	59.9	62.7	.22
Informal	9.2	9.7	8.8	.52
Formal	17.0	17.2	16.7	.79
Religion				
Buddhism	98.3	96.9	99.6	≤.001
Others	1.7	3.1	0.4	≤.001
Household size				
0–4	47.3	53.2	47.7	.70
5 or more	52.7	46.8	52.3	.70
No. of living children				
0	1.2	0.6	1.7	.02
1	35.3	33.7	36.9	.14
2	28.3	30.9	25.6	.01
3 or more	35.2	34.8	35.8	.67
Wealth quintile				
Q1, Poorest	20.9	18.5	23.2	.01
Q2	20.4	18.6	22.1	.05
Q3	20.3	22.5	18.1	.02
Q4	19.7	23.4	16.1	≤.001
Q5, Richest	18.7	17.0	20.5	.05

### Contraceptive Use at Baseline

At baseline in 2011, 71.8% of women surveyed reported not using any form of contraception during the 12 months before the survey (Table 2). Approximately 1 in every 4 women (23.8%) was using a modern method, predominantly a short-acting modern contraceptive (21.3%). Among modern methods, oral pill was the most commonly used, followed by injectables and then condoms. The use of LARCs in the sample was quite low, at 1.7%, and use of permanent methods, even lower, at 0.8%. Among LARCs, IUD use was higher than use of implants. At baseline, no overall significant variation was found in the use of contraceptives between non-voucher areas and areas where the voucher program was about to begin (Table 2).

**TABLE 2.** Contraceptive Use (%) by Type of Contraceptive Method Among Married Women of Reproductive Age, Baseline Survey, 2011

Method	Full Sample (N = 1,936)	Voucher Areas (N = 961)	Non-Voucher Areas (N = 975)	*P* Value
None	71.8	73.7	70.0	.07
Traditional	4.4	3.9	4.8	.35
Modern	23.8	22.4	25.2	.14
Short-acting methods	21.3	20.6	22.1	.44
Pill/emergency pill	11.7	10.6	12.8	.13
Male/female condoms	1.0	0.7	1.2	.26
Injectables	8.6	9.3	8.1	.32
LARCs	1.7	1.4	1.9	.30
IUD	1.1	0.4	1.6	.008
Implants	0.6	1.0	0.3	.08
Permanent methods	0.8	0.4	1.2	.05

Abbreviation: IUD, intrauterine device; LARCs, long-acting reversible contraceptives.

Note the nested nature of the table for the modern method category: the percentages for the pill, condoms, and injectables sum to the short-acting methods percentage while the percentages for the IUD and implants sum to the LARCs percentage. Similarly, the percentages for short-acting methods, LARCs, and permanent methods sum to the modern methods percentage.

In general, the use of short-acting modern methods was uniformly distributed across age groups (Table 3). LARC use, too, although at lower levels, was uniformly distributed across age groups. LARCs were most popular among women of the highest socioeconomic status or with the highest level of education. Permanent methods were most common among the poorest and least educated women and among women with 3 or more children. These patterns mirror those seen in the national Demographic and Health Survey figures for 2010. Overall, these numbers suggest that LARCs may be more accessible and more utilized by women of higher socioeconomic status due to their greater ability to pay and better access to information.

**TABLE 3.** Current Use of Contraceptive Methods (%) by Sociodemographic Characteristics Among Married Women of Reproductive Age, Baseline Survey, 2011

		Type of Method					
	Sample Size (n)	None	Traditional	Modern	Short-Acting	LARCs	PMs
Age group, years							
15–19	47	76.6	0.0	23.4	21.3	2.1	0.0
20–24	428	75.5	2.6	21.9	21.0	0.9	0.0
25–29	615	71.7	4.6	23.7	21.3	1.8	0.6
30–34	453	69.5	4.4	26.1	22.3	2.2	1.6
35–39	229	69.9	6.6	23.5	20.5	1.3	1.7
40–45	164	70.1	6.7	23.2	20.8	1.8	0.6
Education level							
No school	336	72.0	4.2	23.8	21.1	0.9	1.8
Primary	1,139	70.3	3.5	26.2	23.5	1.9	0.8
Secondary	392	75.3	6.9	17.8	16.1	1.5	0.2
High school or higher	69	75.4	5.8	18.8	15.9	2.9	0.0
Wealth quintile							
Q1, poorest	404	67.1	3.7	29.2	26.7	0.8	1.7
Q2	395	71.1	3.8	25.1	22.5	2.0	0.6
Q3	392	70.2	3.8	26.0	23.2	1.8	1.0
Q4	382	73.0	5.5	21.5	19.6	1.6	0.3
Q5, richest	363	78.2	5.2	16.6	13.8	2.2	0.6
No. of living children							
0	23	91.3	0.0	8.7	8.7	0.0	0.0
1	684	78.1	3.7	18.2	17.8	0.4	0.0
2	547	68.6	4.0	27.4	24.5	2.7	0.2
3 or more	682	67.5	5.6	27.0	22.7	2.1	2.2

Abbreviations: LARCs, long-acting reversible contraceptives; PMs, permanent methods.

### Results of Difference-in-Differences Analysis and Multivariate Analyses

In Table 4, we present results of the DID analysis of the use of modern contraceptives in voucher and non-voucher areas. The unadjusted (crude) DID estimates are presented, as well as the associated statistical significance levels for the adjusted DID estimate after controlling for covariates (age, religion, education, occupation, household size, number of living children, participation in other social protection schemes, and socioeconomic status). When contraceptive use is examined by simply disaggregating traditional methods, modern methods, and non-use, no statistically significant DID estimates are observed (Table 4).

**TABLE 4.** Difference-in-Differences Analysis: Change in Use of Contraceptive Methods (%) Between Baseline (2011) and Endline (2013) in Voucher and Non-Voucher Areas

Method	Voucher Areas		Non-Voucher Areas		DID	
	Baseline (n = 961)	Endline (n = 993)	Baseline (n = 975)	Endline (n = 993)	Crude (Unadjusted)	*P* Value (of Adjusted DID Estimate^a^)
None	73.7	63.5	70.0	62.7	−2.9	.41
Traditional	3.9	4.9	4.8	6.3	−0.5	.79
Modern	22.4	31.6	25.2	31.0	3.4	.32
Short-acting	20.6	23.8	22.1	26.7	−1.4	.47
modern						
LARCs	1.4	6.7	1.9	3.5	3.7	.002
Permanent	0.4	1.1	1.2	0.8	1.1	.05
methods						

Abbreviations: DID, difference-in-differences; LARCs, long-acting reversible contraceptives.

a We opted not to present the adjusted DID point estimates with these associated *P* values because the adjusted estimates do not have an intuitive interpretation as the crude estimates do, which are the arithmetic difference-in-differences. Adjusted DID point estimates are available upon request.

Modern contraceptive use increased in both intervention and control areas—in intervention areas, from 22.4% to 31.6%, and in control areas, from 25.2% to 31.0% (Table 4). After disaggregating use of modern contraceptives to examine impacts of the family planning voucher on LARC use, we found that LARC use increased significantly between baseline and endline in both intervention (from 1.4% to 6.7%) and control (from 1.9% to 3.5%) groups (Table 4). More importantly, the increase in the percentage of women using LARCs in voucher areas was greater than the increase in control areas, with an estimated difference in LARC usage rates of 3.7 percentage points (*P* = .002). The statistically significant result on a balanced sample suggests that access to the family planning voucher was associated with a net increase of LARC use among married women. A significant difference was seen also in the increase in use of permanent methods (DID = 1.1 percentage point, *P* = .05).

Use of LARCs increased more in the voucher areas than in the comparison areas.

In Table 5, multivariate analyses of pooled baseline and endline samples present DID estimates after controlling for sociodemographic characteristics. In the logistic regression analysis, the odds ratio for the interaction term between time period (baseline = 0, endline = 1) and area (control = 0, intervention [voucher] = 1) is the effect measure. Adjusted odds ratios from this analysis suggest that, among all married women surveyed, those residing in voucher areas in the intervention period had 1.35 times greater odds of using a modern contraceptive (95% confidence interval [CI], 1.00 to 1.81) than women in control areas or women in the intervention areas during the pre-intervention period (*P* = .05).

**TABLE 5.** Adjusted Odds Ratios From the Logistic Regression Models Predicting Use of Modern Contraceptives, LARCs, and Permanent Methods

Covariates	Use of Modern Methods^a^	*P* Value	Use of LARCs or PMs^b^	*P* Value
	AOR (95% CI)		AOR (95% CI)	
Area (0 = non-voucher; 1 = voucher)	0.73 (0.59, 0.91)	.005	0.55 (0.29, 1.05)	.07
Year (0 = baseline; 1 = endline)	1.24 (1.00, 1.53)	.05	1.11 (0.66, 1.88)	.69
Interaction (area*year)	1.35 (1.00, 1.81)	.05	3.32 (1.54, 7.15)	.002
Age group, years (ref: 15–19)				
20–24	0.87 (0.52, 1.45)	.59	2.25 (0.28, 18.02)	.44
25–29	0.71 (0.42, 1.19)	.19	2.29 (0.28, 18.44)	.44
30–34	0.73 (0.43, 1.24)	.24	1.85 (0.23, 15.22)	.57
35–39	0.70 (0.40, 1.23)	.22	2.16 (0.25, 18.29)	.48
40–45	0.78 (0.43, 1.40)	.41	1.37 (0.16, 12.00)	.78
Education level (ref: no school)				
Primary	1.23 (1.00, 1.52)	.05	0.60 (0.39, 0.95)	.03
Secondary	0.92 (0.71, 1.20)	.54	0.87 (0.49, 1.52)	.62
High school or higher	0.74 (0.48, 1.14)	.17	1.25 (0.50, 3.11)	.64
Employment status (ref: unemployed)				
Agriculture	1.16 (0.96, 1.40)	.13	0.88 (0.57, 1.34)	.54
Informal	1.34 (1.01, 1.78)	.04	1.22 (0.66, 2.26)	.52
Formal	1.49 (1.16, 1.90)	≤.001	1.04 (0.60, 1.80)	.82
No. of living children (ref: 3 or more)				
0	0.08 (0.02, 0.32)	≤.001	No observation	
1	0.54 (0.43, 0.68)	≤.001	0.23 (0.13, 0.43)	≤.001
2	0.96 (0.79, 1.16)	.65	0.51 (0.33, 0.78)	.002
Social health protection (ref: no social health protection program)				
Health Equity Fund	1.87 (1.54, 2.26)	≤.001	1.07 (0.68, 1.69)	.77
Any other social health protection program	1.47 (1.19, 1.82)	≤.001	1.46 (0.91, 2.34)	.11
Wealth quintile (ref: Q1, poorest)				
Q2	0.94 (0.75, 1.17)	.56	0.99 (0.60, 1.65)	.98
Q3	0.98 (0.78, 1.22)	.85	1.16 (0.70, 1.93)	.55
Q4	0.90 (0.71, 1.13)	.35	0.98 (0.57, 1.71)	.95
Q5, richest	0.79 (0.62, 1.02)	.07	1.50 (0.83, 2.74)	.18
Religion (ref: other)	0.65 (0.37, 1.11)	.12	0.61 (0.22, 1.67)	.34
Constant	0.05 (0.01, 0.24)	≤.001	0.20 (0.02, 2.19)	.19

Abbreviations: AOR, adjusted odds ratio; CI, confidence interval; LARCs, long-acting reversible contraceptives; PMs, permanent methods.

a Among all married women in the sample.

b Among those who used any type of contraceptive method in the last 12 months; LARCs and permanent methods grouped together for simplicity of interpretation.

More strikingly, among married women currently using contraceptives, those living in voucher areas in the post-intervention period had 3.3 times greater odds of using a LARC or a permanent method (95% CI, 1.54 to 7.15; *P* = .002) than women in the control groups or in the pre-intervention treatment group. (LARCs and permanent methods are grouped together for ease of comparison in these analyses.) The statistically significant result seen for LARCs and permanent methods increases our confidence in the DID results presented in Table 4 and points to the significant influence of the family planning voucher on the uptake of long-acting methods among married women in Cambodia.

At endline, women living in voucher areas had 3.3 times greater odds than women in non-voucher areas of using a LARC or a permanent method than women in the control groups or in the pre-intervention voucher group.

### LARC Use Before and After Intervention

In disaggregated analysis of LARC use, we found that the greater increases in LARC uptake in voucher areas than in control areas took place across age groups, educational status, occupational groups, and socioeconomic levels (Table 6). The greatest increases were seen among women with the lowest levels of education (no school, from 1.1% at baseline to 11.8% at endline) and in the lowest socioeconomic group (poorest quintile, from 1.1% to 8.8%). These results suggest that the family planning voucher increased use of LARCs most among the poorest and most vulnerable women, the intended beneficiaries of the intervention. Another key finding is that large increases in the use of LARCs occurred among women with 3 or more children (from 1.8% to 11.0%), suggesting that a provider bias existed and/or that LARCs may be more appealing to women who have already reached their desired family size.

**TABLE 6.** Changes in Use of LARCs (%) Between Baseline (2011) and Endline (2013) by Selected Characteristics

	Voucher Areas		Non-Voucher Areas	
	Baseline (n = 961)	Endline (n = 993)	Baseline (n = 975)	Endline (n = 993)
All married women	1.4	6.7	1.9	3.5
Age group, years				
15–19	0.0	0.0	4.3	0.0
20–24	5.4	4.7	0.8	2.8
25–29	6.7	6.3	1.9	4.1
30–34	7.0	7.0	2.3	3.3
35–39	9.3	9.1	3.1	3.6
40–45	8.8	8.6	2.1	6.4
Education level				
No school	1.1	11.8	0.6	4.8
Primary	0.9	6.0	2.8	2.9
Secondary	2.2	5.6	0.9	4.5
High school or higher	5.3	5.8	0.0	3.2
Wealth quintile				
Q1, poorest	1.1	8.8	0.4	3.1
Q2	1.7	7.9	2.3	3.4
Q3	0.9	5.1	2.8	4.3
Q4	0.4	6.5	3.2	2.9
Q5, richest	3.0	5.1	1.5	3.9
No. of living children				
0	0.0	0.0	0.0	0.0
1	0.3	3.3	0.6	1.8
2	2.0	7.3	3.6	4.1
3 or more	1.8	11.0	2.3	5.5

Abbreviation: LARCs, long-acting reversible contraceptives.

## DISCUSSION

These results demonstrate the ability of a family planning voucher program to increase uptake of long-acting methods among poor women in Cambodia, contribute to the body of evidence on the impact of vouchers on LARC uptake. The impact seen on LARC uptake in this study shows the potential for demand-side strategies such as vouchers to complement supply-side and policy-level efforts to increase voluntary uptake and expand choice among poor and vulnerable women to include effective long-acting methods. This is particularly important since access to family planning is among the most inequitably distributed of reproductive health indicators in most low- and middle-income countries.

The demonstrated positive effect of the Cambodian family planning voucher program is indicative of an effective, comprehensive, and targeted implementation strategy. The voucher program in Cambodia targets not only financial barriers but also informational barriers that disproportionately impede poor and marginalized women from making informed contraceptive choices. The largest gains in LARC uptake occurred among women from the lowest socioeconomic strata. This suggests that vouchers may be an effective strategy for giving access to LARCs to women who might be unable to obtain the methods they want without the help of a voucher. For poor women in Cambodia, initial cost appears to be a barrier to the choice of LARCs and permanent methods—a barrier that vouchers can lower.

The voucher program in Cambodia targets financial and information barriers that disproportionately impede poor and marginalized women from making informed contraceptive choices.

### Limitations

There are limitations to this study. Assessment of current contraceptive use measured in cross-sectional surveys has limited ability to deal with method discontinuation and switching, particularly for short-acting methods. This limitation may be less problematic for LARCs, however, which have lower rates of discontinuation. Additionally, although our study attempted to address confounding influences from observed covariates through the quasi-experimental design, there is a possibility that, despite a stringent matching design to generate balanced pre-intervention samples, spurious associations due to unobserved confounders could be present. These confounders could include contamination or exposure to other non-public social health protection or other nationwide system improvements that resulted in better supply chain management and improved referral systems and execution of programs if they were not uniform between control and intervention groups at baseline. We did not collect information on other demand promotion programs that may have been carried out outside the public sector. Lastly, it is not possible to disaggregate the effects of the vouchers themselves and the voucher-associated promotion activities. Moreover, the execution of the intervention may have had a positive impact on the outcome over and above the voucher program itself. While some activities and attention occurred in both the voucher and non-voucher areas, the various activities such as training, supervision, and other attention from the research team to implement the voucher intervention may have contributed to improving service delivery and the outcome.

## CONCLUSION

Despite these limitations, the impacts observed in this study of family planning vouchers are significant for two key reasons. First, this is the first published study of the Cambodian family planning voucher strategy, and it finds that vouchers increased LARC uptake among important beneficiary groups. Second, and more importantly, these results lay the groundwork for rigorously generating evidence on demand-side strategies aimed at improving the capacity of poor and vulnerable women in low- and middle-income countries to make and carry out informed decisions about contraceptive use. Such strategies should expand women’s choice and agency, in line with a rights-based understanding of contraceptive service delivery. This study also has generated lessons for national family planning programs that seek to expand contraceptive choice and improve equity in access to effective long-acting methods. The findings may serve as an impetus to integrate strategies such as vouchers into larger national family planning initiatives.

## Notes

**Transferred Peer Review**
